# Contrasting In Vitro Activity of Nitroxoline Against Multidrug-Resistant *Escherichia coli* and *Klebsiella pneumoniae* Isolates from Outpatients

**DOI:** 10.3390/antibiotics15050479

**Published:** 2026-05-08

**Authors:** Dominik Maleš, Zvonimir Barišić, Darko Kero, Merica Carev

**Affiliations:** 1 Teaching Institute for Public Health, Split-Dalmatia County, 21000 Split, Croatia; 2 General Hospital of Šibenik-Knin County, 22000 Sibenik, Croatia; 3 Study Program of Dental Medicine, School of Medicine, University of Split, 21000 Split, Croatia; darko.kero@mefst.hr; 4 University of Split, Faculty of Health Sciences, 21000 Split, Croatia; 5 School of Medicine, University of Split, 21000 Split, Croatia; 6 ESCMID Food-and-Waterborne Infections Study Group, 4051 Basel, Switzerland

**Keywords:** antibacterial agents, antimicrobial susceptibility testing, antibiotic resistance screening, *E. coli*, *K. pneumoniae*, multidrug-resistant bacteria, nitroxoline

## Abstract

**Background/Objectives:** Antimicrobial resistance is a major public health concern, particularly among multidrug-resistant (MDR) *Enterobacterales*. Nitroxoline is an older antibiotic with a unique mechanism of action that has not been widely used in recent decades. This study aimed to evaluate the in vitro activity of nitroxoline against Extended-Spectrum Beta-Lactamase (ESBL)-producing *Escherichia coli* (*E. coli*) and MDR *Klebsiella pneumoniae* (*K. pneumoniae*) isolated from urine samples of outpatients in Split-Dalmatia County, Croatia. **Methods:** Nitroxoline susceptibility was assessed using disk diffusion for *E. coli* (n = 26) and broth microdilution for *K. pneumoniae* (n = 33). **Results:** ESBL-producing *E. coli* isolates showed high susceptibility to nitroxoline, with 25 (96.2%) classified as susceptible. In contrast, *K. pneumoniae* isolates exhibited high minimum inhibitory concentration (MIC) values (range 16–128 mg/L; median 32 mg/L), indicating limited activity. **Conclusions:** Nitroxoline demonstrated strong in vitro activity against ESBL-producing *E. coli* but limited activity against MDR *K. pneumoniae*. Interpretation of the findings for *K. pneumoniae* is constrained by the lack of established clinical breakpoints.

## 1. Introduction

*Enterobacterales*, including *Escherichia coli* and *Klebsiella pneumoniae*, are important pathogens that frequently exhibit multidrug resistance (MDR), defined as non-susceptibility to at least one agent in three or more antimicrobial categories [1,2]. Antimicrobial resistance is a major global public health issue associated with increased morbidity and mortality due to treatment failure [3,4,5,6,7].

Third-generation cephalosporin-resistant *E. coli* and carbapenem-resistant *K. pneumoniae* are among the most significant pathogens burdening healthcare systems in Europe [4]. The increasing prevalence of Extended-Spectrum Beta-Lactamase (ESBL)-producing *Enterobacterales* has led to greater reliance on carbapenems, which in turn has contributed to the emergence of carbapenem-resistant strains [2,8,9,10,11]. A significant increase in carbapenem-resistant *K. pneumoniae* has been observed across Europe, including Croatia, where rising resistance further limits available treatment options [4,12,13,14,15]. However, data on antimicrobial resistance among outpatient isolates remain limited.

Therefore, there is a need to explore alternative treatment options, including the repurposing of older antibiotics such as nitroxoline [16,17]. Nitroxoline is an oral antimicrobial agent historically used for the treatment of urinary tract infections, particularly those caused by *E. coli*. Despite demonstrated in vitro activity, its broader clinical use has been limited to certain European countries [17,18,19,20]. Key limitations include the lack of modern randomized clinical trials, limited pharmacokinetic and pharmacodynamic data, and restricted regulatory approval [17,21,22,23]. Its antimicrobial activity is thought to involve metal ion chelation and subsequent disruption of bacterial metabolism with the reduction of transmembrane potential, overaccumulation of reactive oxygen species and interference with the electron transport chain [24,25].

Previous studies have demonstrated strong in vitro activity of nitroxoline against non-multidrug-resistant (MDR) uropathogens, with MIC values below established clinical breakpoints for *E. coli* [26,27]. However, data on its effectiveness against MDR isolates, particularly from outpatient settings, remain limited [24,26,28]. In Croatia, data on nitroxoline susceptibility are scarce, and, to our knowledge, its activity against carbapenemase-producing *K. pneumoniae* has not been previously investigated.

There is a need for further research to better define the role of nitroxoline in the treatment of infections caused by MDR *Enterobacterales*, particularly *K. pneumoniae*, for which clinical breakpoints are not established [20,29]. This study aimed to evaluate the in vitro activity of nitroxoline against MDR *E. coli* and *K. pneumoniae* isolates from outpatients, with a particular focus on ESBL-producing *E. coli* and carbapenem-resistant *K. pneumoniae*.

## 2. Results

### 2.1. Bacterial Strains Collection

Urine samples from outpatients were collected across Split-Dalmatia County and processed at the Teaching Institute for Public Health of Split-Dalmatia County (TIPH) in Split, Croatia, between 1 November 2023, and 1 May 2024. All patients were outpatients with urinary tract infections, and duplicate isolates from the same individual were excluded using unique patient identifiers.

A total of 5152 single-patient urine samples, each representing a unique infection, were collected during the study period. Of these, 2.25% were culture-positive for MDR *E. coli* or *K. pneumoniae*. A total of 66 consecutive MDR isolates were further analyzed, including 27 ESBL-producing *E. coli*, 8 ESBL-producing *K. pneumoniae*, 28 OXA-48-producing *K. pneumoniae*, and 3 KPC-producing *K. pneumoniae*. As subcultivation of three isolates (two OXA-48-producing and one KPC-producing *K. pneumoniae*) was unsuccessful, the final sample included 63 isolates.

### 2.2. Demographic Characteristics of Patients 

A total of 63 bacterial isolates were collected: 29 (46%) from men and 34 (54%) from women, with no statistically significant difference in distribution (χ^2^ = 0.79; *p* = 0.373). Patient age ranged from 19 to 97 years (median 76; IQR 19). Most isolates were obtained from patients over 65 years of age (n = 46, 73%), followed by 11 (17%) from the 45–64 age group, 5 (8%) from the 25–44 group, and 1 (2%) from patients aged ≤24 years. The proportion of patients over 65 was significantly higher than that of all other age groups combined (χ^2^ = 26.7; *p* < 0.001).

### 2.3. Antibiotic Susceptibility of E. coli

**Figure 1:**
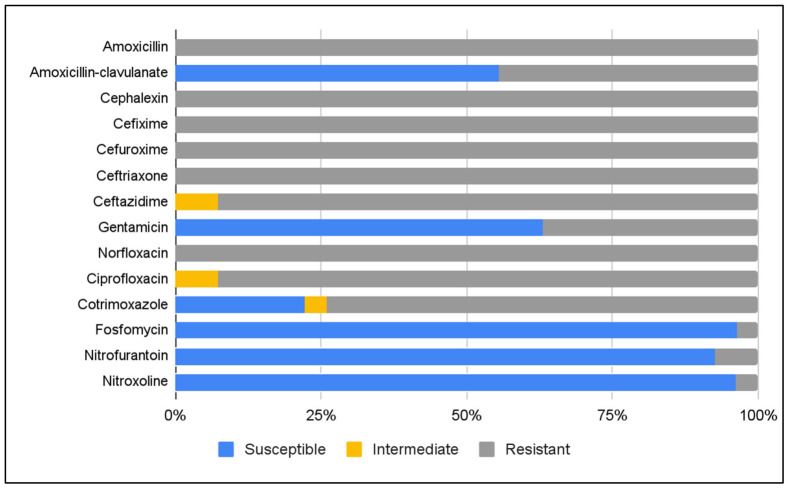
Susceptibility of ESBL-producing *E. coli* strains to routinely tested antibiotics and nitroxoline.

Susceptibility of 27 ESBL-producing *E. coli* isolates was assessed by disk diffusion against a panel of commonly used antibiotics (Figure 1) [29,30]. All isolates (100%) were resistant to amoxicillin, cephalexin, cefixime, cefuroxime, ceftriaxone, and norfloxacin. Resistance rates were also high for ceftazidime (92.6%) and ciprofloxacin (92.6%), and lower for cotrimoxazole (74.1%). Resistance to amoxicillin–clavulanate was observed in 44.4% of isolates, and to gentamicin in 37%. High susceptibility was observed for fosfomycin and nitrofurantoin, with 26 (96.3%) and 25 (92.6%) susceptible isolates, respectively (Figure 1).

### 2.4. Antibiotic Susceptibility of K. pneumoniae

**Figure 2:**
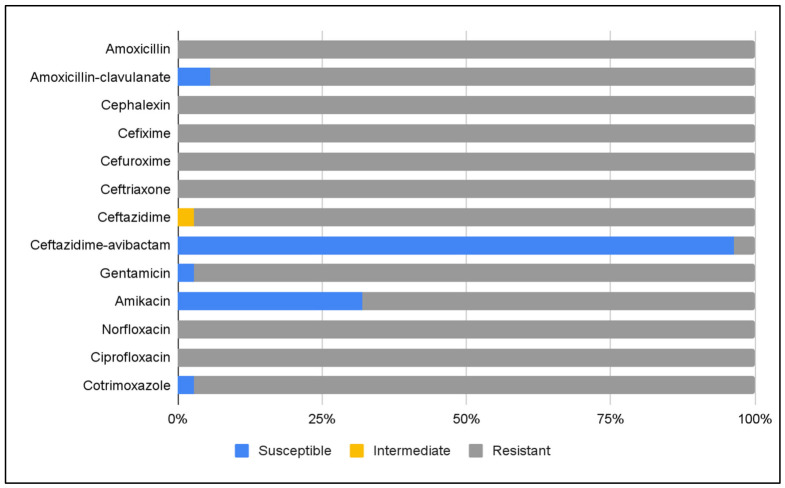
Susceptibility of ESBL- and carbapenemase-producing *K. pneumoniae* strains to routinely tested antibiotics.

Susceptibility of 36 ESBL- and carbapenemase-producing *K. pneumoniae* isolates was assessed by disk diffusion against a panel of commonly used antibiotics. Susceptibility to ceftazidime–avibactam and amikacin was additionally tested in 28 (77.8%) and 25 (69.4%) isolates, respectively. All isolates (100%) were resistant to amoxicillin, cephalexin, cefixime, cefuroxime, ceftriaxone, norfloxacin, and ciprofloxacin. Resistance rates were also high for amoxicillin–clavulanate (94.4%), ceftazidime (97.2%), gentamicin (97.2%), and cotrimoxazole (97.2%), with lower resistance to amikacin (68%). High susceptibility was observed for ceftazidime–avibactam, with 27 (96.4%) susceptible isolates (Figure 2).

**Figure 3:**
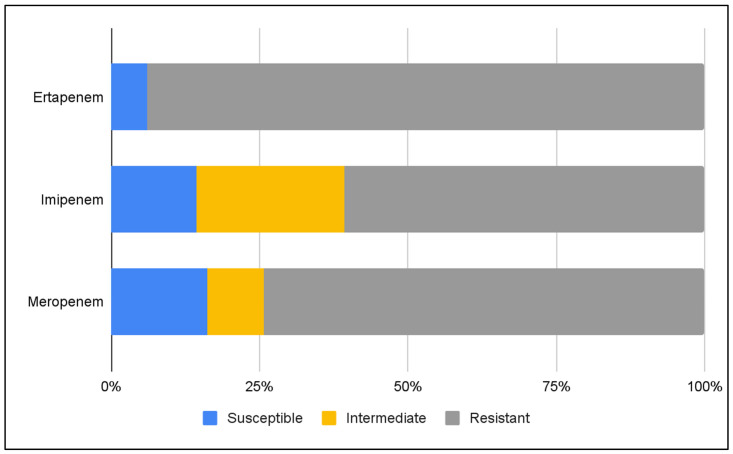
Susceptibility of ESBL- and carbapenemase-producing *K. pneumoniae* strains to carbapenems.

Susceptibility to carbapenems was assessed in a subset of isolates. Ertapenem was tested in 33 (91.7%) isolates, with 31 (93.9%) showing resistance. Imipenem was tested in 28 (77.8%) isolates, with 17 (60.7%) being resistant, while meropenem was tested in 31 (86.1%) isolates, with 23 (74.2%) being resistant (Figure 3).

Resistance patterns in *E. coli* and *K. pneumoniae* were largely similar, with both species showing high resistance to most β-lactam antibiotics, fluoroquinolones, and cotrimoxazole. A statistically significant difference between the two species was observed only for amoxicillin–clavulanate, gentamicin, and cotrimoxazole (*p* < 0.001). Since resistance to most other tested antibiotics was 100% in both species, further statistical comparisons were not informative.

### 2.5. Susceptibility to Nitroxoline

Susceptibility of 26 ESBL-producing *E. coli* isolates to nitroxoline was assessed by disk diffusion according to EUCAST guidelines [29,30]. All but one isolate were susceptible, corresponding to a susceptibility rate of 96.2% (Figure 1).

**Figure 4:**
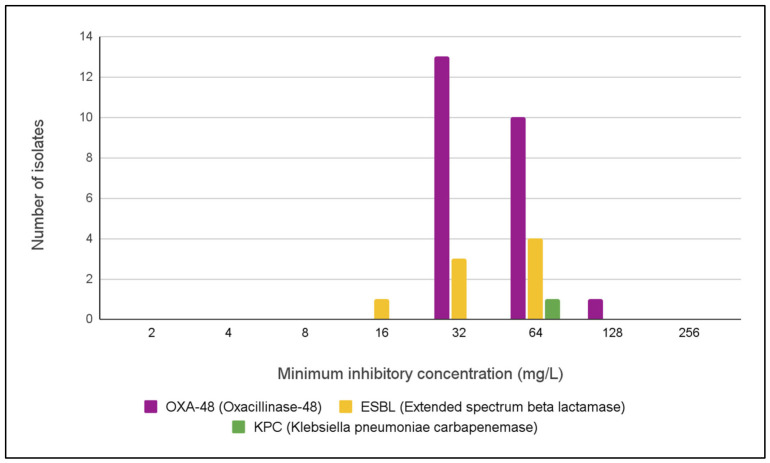
Minimum inhibitory concentration (MIC) of nitroxoline against OXA-48, ESBL-, and KPC-producing *K. pneumoniae* isolates.

Of the 36 collected *K. pneumoniae* isolates, three (one KPC- and two OXA-48-producing strains) were excluded due to unsuccessful subcultivation. The remaining 33 isolates were tested using the broth microdilution method (Clinical and Laboratory Standards Institute (CLSI) [31]), and results are expressed as minimum inhibitory concentrations (MICs, mg/L) (Figure 4). MIC values ranged from 16 to 128 mg/L, with a median of 32 mg/L and a mean of 48.97 mg/L.

## 3. Discussion

The high susceptibility rate (96.2%) of ESBL-producing *E. coli* to nitroxoline indicates that this agent may represent a potential treatment option for uncomplicated urinary tract infections caused by these strains. In contrast, the high MIC values observed for MDR *K. pneumoniae* suggest limited activity of nitroxoline against these isolates. However, interpretation of these findings is constrained by the absence of established clinical breakpoints for *K. pneumoniae* (Figure 4).

Treatment options for infections caused by MDR *Enterobacterales* remain limited, particularly in outpatient settings. In our study, ceftazidime–avibactam was the only β-lactam antibiotic showing high activity against MDR *K. pneumoniae* (Figure 2). Compared to MDR *E. coli*, *K. pneumoniae* isolates exhibited significantly higher resistance rates to amoxicillin–clavulanate, gentamicin, and cotrimoxazole, further emphasizing the limited therapeutic options for these infections.

*E. coli* isolates showed high susceptibility to nitrofurantoin and fosfomycin, which is consistent with their established role in the treatment of uncomplicated urinary tract infections [32]. High susceptibility of *K. pneumoniae* to ceftazidime–avibactam was also observed. Resistance rates for most other antibiotics were higher than previously reported, likely reflecting the selection of MDR isolates in this study. The distribution of carbapenemase-producing *K. pneumoniae* isolates in our study is consistent with previously reported patterns in Croatia, suggesting overlap between community and healthcare-associated strains [33,34].

These findings are consistent with previous studies reporting high susceptibility of *E. coli*, including MDR strains, to nitroxoline [17,18]. Some in vivo studies have confirmed the efficacy of nitroxoline in treating uncomplicated UTIs. In a literature review of 28 studies involving over 11,000 patients, Naber et al. found that nitroxoline was effective in eliminating bacteriuria in patients with uncomplicated UTIs while maintaining a high safety profile [23]. Although these results support the potential use of nitroxoline for the treatment of uncomplicated urinary tract infections caused by *E. coli*, its clinical application remains limited by the lack of modern randomized clinical trials, insufficient pharmacokinetic and pharmacodynamic data, and restricted regulatory approval [17,21,22,23]. These factors, together with its primarily urinary activity and limited systemic use, may explain why nitroxoline is not widely used in current clinical practice.

The activity of nitroxoline against MDR *K. pneumoniae* in our study was characterized by relatively high MIC values (Figure 4). To our knowledge, this is the first report of such data from Croatia. In contrast to our findings, previous studies have generally reported lower MIC values for carbapenemase-producing *K. pneumoniae* isolates [35,36,37]. This discrepancy may reflect differences in isolate selection, resistance profiles, or local epidemiology.

The absence of established clinical breakpoints for nitroxoline in *K. pneumoniae* limits the interpretation of MIC results and prevents reliable classification of susceptibility. Although breakpoints defined for *E. coli* have been applied in some studies, their use for other species remains uncertain [29,36,37,38]. Therefore, the relatively high MIC values observed in this study should be interpreted with caution, highlighting the need for standardized interpretive criteria. The relatively small number of isolates in this study may limit the generalizability of the findings. Nevertheless, our results provide additional insight into the activity of nitroxoline against MDR *Enterobacterales* in outpatient settings. Further studies with larger sample sizes are needed to better define the activity of nitroxoline against MDR *K. pneumoniae* and to support the development of standardized susceptibility testing and clinical breakpoints [16,29,35,36,39]. 

The underlying resistance mechanisms of MDR *K. pneumoniae* were not investigated in this study. Further research is needed to clarify the role of nitroxoline in the treatment of infections caused by MDR *Enterobacterales*.

## 4. Materials and Methods

### 4.1. Strains, Culture Conditions, Antibiotics, and Chemicals

*Enterobacterales* were isolated from midstream morning urine samples using standard microbiological procedures. Briefly, 10 µL of urine was inoculated onto selective UTI chromogenic agar (Oxoid Limited, Basingstoke, UK) and incubated at 37 °C for 24 h. Bacterial identification was performed using API 10 S biochemical tests (bioMérieux, Marcy-l’Étoile, France). Antimicrobial susceptibility testing was conducted using the Kirby–Bauer disk diffusion method and interpreted according to EUCAST breakpoints [29,30]. A total of 63 MDR *E. coli* and *K. pneumoniae* isolates were included in the study.

### 4.2. Disk Diffusion Test

Disk diffusion was used for *E. coli* in accordance with EUCAST guidelines, for which interpretive breakpoints are available, whereas for *K. pneumoniae*, where no established breakpoints exist, susceptibility was assessed using the broth microdilution method and expressed as MICs. The susceptibility of *E. coli* strains to nitroxoline was tested using the disk diffusion method, as outlined in EUCAST guidelines [29,30]. A sterile swab was used to touch the tip of a selected colony, which was then mixed into a test tube containing sterile saline. The resulting suspension was adjusted to a turbidity of 0.5 McFarland standard using a photometric device (bioMérieux, Marcy-l’Étoile, France). The suspension was spread evenly over the surface of Mueller–Hinton agar (MHA, Biolife Italiana, Monza, Italy). Antimicrobial susceptibility disks (Mast Diagnostica, Reinfeld, Germany) were added, and the plates were incubated at 37 °C for 24 h under aerobic conditions. The inhibition zones were interpreted according to EUCAST breakpoints [29]. Breakpoints for the interpretation of inhibition zone diameters are available for nitroxoline (15/15 mm) (Mast Diagnostica, Reinfeld, Germany), but they apply exclusively to *E. coli* isolates. There are no defined breakpoints for accurate interpretation of the disk diffusion test for *K. pneumoniae* [29].

### 4.3. Double Disc Synergy Test (DDST)

ESBL production was detected using a phenotypic combined disk method. Disks containing ceftazidime (30 µg) and cefotaxime (30 µg) (Mast Diagnostica, Reinfeld, Germany) were placed on Mueller–Hinton agar (MHA; Biolife Italiana, Monza, Italy) in proximity to a disk containing amoxicillin–clavulanic acid (20/10 µg)( Mast Diagnostica, Reinfeld, Germany). The distance between disks was 20 mm. A positive result was defined as an increased inhibition zone around either cephalosporin disk or the presence of a characteristic “keyhole” effect toward the amoxicillin–clavulanic acid disk [40].

### 4.4. Detection of Carbapenemases

All enterobacterial isolates with reduced susceptibility to at least one carbapenem by the disk diffusion test were further tested for carbapenemase production. The Coris Resist-5 O.K.N.V.I test (Coris BioConcept, Gembloux, Belgium), an immunochromatographic assay for detecting carbapenemase production in *Enterobacterales*, was used. This test detects OXA-48, KPC, NDM, VIM, and IMP carbapenemases. To perform the test, twelve drops of the provided buffer were added to a test tube. Using a sterile swab, one colony of the tested strain was picked from the agar plate and immersed in the test tube, then mixed using a vortex. Three drops of the sample were placed on the sample pad, and after 15 min, the test was ready for interpretation. The presence of both a positive control line and a positive test line indicates that the tested strain produces the corresponding carbapenemase.

### 4.5. Determining the Minimum Inhibitory Concentrations of K. pneumoniae Strains

The susceptibility of *K. pneumoniae* strains to nitroxoline was tested using the broth microdilution method following CLSI guidelines and Cherdtrakulkiat [26,31]. Since clinical breakpoints for nitroxoline apply solely to *E. coli* isolates, EUCAST recommends broth microdilution as the reference method for antimicrobial susceptibility testing of aerobic bacteria, with MICs serving as the basis for interpretation [41]. Nitroxoline powder (Merck, Darmstadt, Germany) was dissolved in dimethyl sulfoxide (DMSO, Merck, Darmstadt, Germany) to obtain a stock solution of 1024 μg/mL. Serial twofold dilutions were prepared in Mueller–Hinton broth (MHB) to achieve concentrations ranging from 512 to 2 μg/mL. Aliquots (50 μL) of each concentration were dispensed into 96-well microplates.

Bacterial inoculum was prepared from overnight cultures and adjusted to a turbidity equivalent to 0.5 McFarland standard using a photometric device. Microplate wells were inoculated with 50 μL of the bacterial suspension and incubated at 35 ± 2 °C for 16–20 h. Each microplate included a positive control (bacterial suspension only) and a negative control (MHB only). The final concentration of DMSO in the test wells was minimized and maintained at a level not expected to affect bacterial growth. A solvent control was included to exclude any potential effect of DMSO. MIC determinations were performed once for each isolate. The minimum inhibitory concentration (MIC) was defined as the lowest concentration of nitroxoline that inhibited visible bacterial growth. Mueller–Hinton broth (MHB; Biolife Italiana, Monza, Italy), a cation-adjusted medium compliant with ISO 20776-1 standards [42], was used for all assays [43]. The pH of each batch was 7.3, and this was verified prior to testing; quality control was performed using an American Type Culture Collection (ATCC) *E. coli* strain.

Although divalent cations such as Ca^2+^ and Mg^2+^ may influence the activity of certain antibiotics, their impact on nitroxoline has not been clearly demonstrated. Nitroxoline preferentially chelates transition metals such as zinc, iron, and copper while showing limited interaction with calcium and magnesium [25,26]. Given the low zinc content in Mueller–Hinton broth, a significant effect of medium composition on MIC values is considered unlikely.

### 4.6. Statistical Analysis

Descriptive statistics were used to summarize minimum inhibitory concentrations (MICs), resistance frequencies, and patient characteristics. Differences between groups were assessed using the chi-square (χ^2^) test, including comparisons of resistance rates between *E. coli* and *K. pneumoniae* for selected antibiotics (amoxicillin–clavulanate, gentamicin, and cotrimoxazole), as well as differences in patient distribution by gender and age groups. Statistical significance was set at α = 0.05. Analyses were performed using CalculatorSoup (Furey Edward, Ashland, MA, USA. Available online at https://www.calculatorsoup.com (accessed on 13 April 2026)), Microsoft Excel (v16.0) (Microsoft Corporation, Redmond, WA, USA), and VassarStats (Richard Lowry, Poughkeepsie, NY, USA. Available online at http://vassarstats.net/ (accessed on 13 April 2026)).

## 5. Conclusions

Nitroxoline demonstrated strong in vitro activity against ESBL-producing *E. coli*, supporting its potential use in the treatment of uncomplicated urinary tract infections. In contrast, high MIC values observed for ESBL- and carbapenemase-producing *K. pneumoniae* suggest limited activity against this pathogen. Interpretation of these findings is constrained by the lack of established clinical breakpoints for *K. pneumoniae*. Further studies are needed to better define the role of nitroxoline in the treatment of infections caused by MDR *Enterobacterales*.

## Data Availability

The original contributions presented in this study are included in the article/Supplementary Material. Further inquiries can be directed at the corresponding authors.

## Supplementary Materials

The following supporting information can be downloaded at: https://www.mdpi.com/article/10.3390/antibiotics15050479/s1, Table S1: The minimum inhibitory concentration (MIC) of nitroxoline for *K. pneumoniae* isolates. The X denotes strains with failed subcultivation.
