# Supplementary material for: Contrasting In Vitro Activity of Nitroxoline Against Multidrug-Resistant Escherichia coli and Klebsiella pneumoniae Isolates from Outpatients
Source: Antibiotics (Basel). 2026 May 8;15(5):479. doi: 10.3390/antibiotics15050479 (PMC13203481; doi:10.3390/antibiotics15050479)
Supplement: Supplementary file 1 [file antibiotics-15-00479-s001.zip › antibiotics-4213286-supplementary.pdf]

Supplementary Material

Table S1. The minimum inhibitory concentration (MIC) of nitroxoline for *K. pneumoniae* isolates. X denotes strains with failed subcultivation.

Designated strain number	*Beta-lactamase*	*Minimum inhibitory concentration (mg/L)*
2350973	OXA-48	64
2351765	OXA-48	X
2351777	ESBL	32
2352254	OXA-48	64
2352887	KPC	X
2353509	OXA-48	64
2353693	OXA-48	32
2353739	OXA-48	32
2354239	OXA-48	32
2355781	OXA-48	32
2355790	OXA-48	64
2355849	OXA-48	64
2355914	OXA-48	32
2356272	ESBL	64
2356565	OXA-48	64
2356568	OXA-48	32
2356594	ESBL	64
2356716	ESBL	32
2356780	OXA-48	32
2357130	OXA-48	32
2359130	OXA-48	64
2359167	OXA-48	32
2360105	OXA-48	32
2360759	ESBL	64
2360821	OXA-48	X
2360948	KPC	64
2361532	ESBL	32
2361578	OXA-48	32
2364358	OXA-48	64
2364646	OXA-48	64
2366764	OXA-48	64
2367332	ESBL	64
2367374	ESBL	16
2400577	OXA-48	128
2403125	OXA-48	32
2403596	OXA-48	32
